# Landscape of Germline Mutations in DNA Repair Genes for Breast Cancer in Latin America: Opportunities for PARP-Like Inhibitors and Immunotherapy

**DOI:** 10.3390/genes10100786

**Published:** 2019-10-10

**Authors:** Laura Keren Urbina-Jara, Augusto Rojas-Martinez, Emmanuel Martinez-Ledesma, Dione Aguilar, Cynthia Villarreal-Garza, Rocio Ortiz-Lopez

**Affiliations:** 1 Tecnologico de Monterrey, Escuela de Medicina y Ciencias de la Salud, Monterrey 64710, Mexico; 2 Tecnologico de Monterrey, Centro de Cancer de Mama, Hospital Zambrano Hellion, San Pedro Garza Garcia 66278, Mexico; 3 Instituto Nacional de Cancerologia, Departamento de Investigacion, Av. San Fernando #22, Tlalpan, Ciudad de Mexico 14080, Mexico

**Keywords:** breast cancer, BRCA1, BRCA2, DNA repair, Latin America, germline, PARP inhibitors therapy

## Abstract

Germline mutations in *BRCA1* and *BRCA2* (*BRCA1/2*) genes are present in about 50% of cases of hereditary breast cancer. Proteins encoded by these genes are key players in DNA repair by homologous recombination (HR). Advances in next generation sequencing and gene panels for breast cancer testing have generated a large amount of data on gene variants implicated in hereditary breast cancer, particularly in genes such as *PALB2*, *ATM*, *CHEK2*, *RAD51*, *MSH2*, and *BARD1*. These genes are involved in DNA repair. Most of these variants have been reported for Caucasian, Jewish, and Asian population, with few reports for other communities, like those in Latin American (LA) countries. We reviewed 81 studies from 11 LA countries published between 2000 and 2019 but most of these studies focused on *BRCA1/2* genes. In addition to these genes, breast cancer-related variants have been reported for *PALB2*, *ATM*, *CHEK2*, *BARD1*, *MLH1*, *BRIP1*, *MSH2*, *NBN*, *MSH6,* and *PMS2* genes. Some of these variants are unique to LA populations. This analysis may contribute to enhance breast cancer variant characterization, and thus to find therapies and implement precision medicine for LA communities.

## 1. Introduction

Breast cancer is the leading cause of cancer death in women worldwide, with about 627,000 deaths in 2018 [1]. About 5–10% of all breast cancer is caused by germline variants in *BRCA1/2* [2,3]. Moreover, about 50% of hereditary breast cancer (HBC) cases present germline mutations in *BRCA* genes [2]. *BRCA1/2* pathogenic variants confer more than 50% risk of breast cancer development [4]. Since the identification of breast cancer genes *BRCA1/2* in 1994 and 1995, respectively [5,6,7], a large amount of data on the risks conferred by these genes for breast and other cancers has been generated. These genes play an important role in the repair of double-strand breaks in DNA by homologous recombination (HR) along with a plethora of additional proteins [2,8,9,10]. 

About 50–60% of cases of HBC show variants in *BRCA1/2* genes. The remaining percentage involves moderate and low penetrance variants in non-BRCA genes [11]. Next generation sequencing (NGS) and other technologies are still identifying variants in genes associated with breast cancer. Non-BRCA variants have been observed in genes like *ATM*, *PALB2*, *RAD51*, and *BARD1* [4,12,13]. Like BRCA genes, these genes codify for proteins participating in DNA repair [14,15]. 

The National Comprehensive Cancer Network (NCCN) guidelines for genetic assessment in hereditary breast cancer (version 3.2019) recommends genetic evaluation of *ATM*, *CDH1*, *CHEK2*, *NBN*, *NF1*, and *PALB2* in addition to *BRCA1/2* genes. There is accumulative evidence that variants in *BARD1*, *BRIP1*, *MSH2*, *MLH1*, *MSH6*, *PMS2*, *RAD51C*, and *RAD51D* are also causative of HBC, although some of these gene products participate in other DNA repair pathways and there is insufficient evidence for establishing management strategies [16]. This generates a paradigm for genetic counseling and assessment. Based on the absence of functional studies evaluating the activity of these genes, genetic advisory is being challenged by patient management. 

Notably, most of the data on HBC variants represent Caucasian populations [17]. HBC accounts for about 15% of breast cancer cases in Latin America (LA) [18]. This population, resulting from the combination of Native American, Spanish, African, and other communities, is highly heterogeneous, even within the regions of the different countries where they live [19,20,21]. This diversity impacts the genetic variation involved in HBC in this large community [20]. Unfortunately, the data on HBC variants for this population is scarce and mainly focused on *BRCA1/2*; few cumulative reports on these variants have been reported. This information will be required to implement programs of precision medicine for HBC patients belonging to this population, like those that are being implemented for prevention and therapy of *BRCA1/2* variants, particularly with Poly-ADP ribose polymerase (PARP) inhibitors [22,23,24,25,26,27]. Additionally, studies are suggesting that some tumor types with mutations in DNA repair genes are amenable for immunotherapy approaches [28,29,30,31,32,33]. This review analyzes 81 studies of germline mutations in breast cancer from 11 LA countries published between 2000 and 2019, with the focus on BRCA and non-BRCA genes involved in DNA repair.

## 2. Materials and Methods 

**Figure 1:**
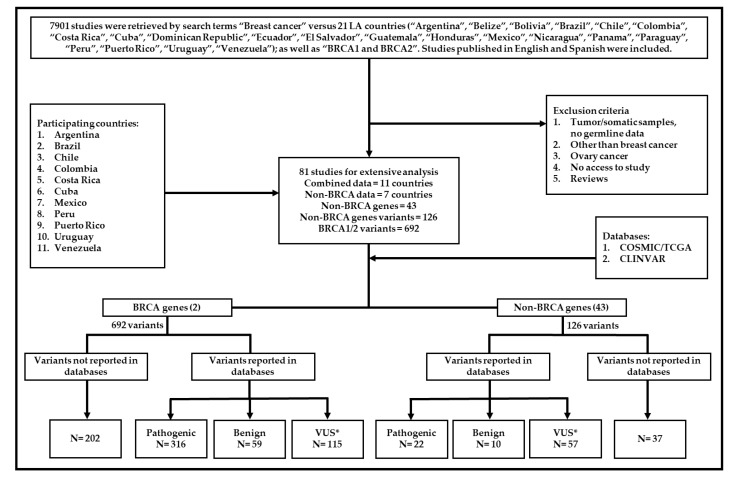
Study diagram. This diagram shows data acquisition and variant classification. *VUS: conflicting interpretation, uncertain significance, likely pathogenic, likely benign, risk factor, drug response.

We searched the PubMed database (https://www.ncbi.nlm.nih.gov/pubmed/) for all breast cancer studies in 21 LA countries. Studies published between 2000 and April 5, 2019 were considered. A total of 7901 studies were retrieved using the following search terms “Breast cancer” and 21 LA countries (“Argentina”, “Belize”, “Bolivia”, “Brazil”, “Chile”, “Colombia”, “Costa Rica”, “Cuba”, “Dominican Republic”, “Ecuador”, “El Salvador”, “Guatemala”, “Honduras”, “Mexico”, “Nicaragua”, “Panama”, “Paraguay”, “Peru”, “Puerto Rico”, “Uruguay”, and “Venezuela”); as well as “*BRCA1* and *BRCA2*”. Studies published in English and Spanish were included. Titles and abstracts were reviewed, and ineligible reports were discarded. The inclusion criteria considered research papers, case reports, and germline mutation data. A diagram showing the process of data acquisition is presented in Figure 1. 

Considering the inclusion criteria, 81 studies were selected for extensive analysis [34,35,36,37,38,39,40,41,42,43,44,45,46,47,48,49,50,51,52,53,54,55,56,57,58,59,60,61,62,63,64,65,66,67,68,69,70,71,72,73,74,75,76,77,78,79,80,81,82,83,84,85,86,87,88,89,90,91,92,93,94,95,96,97,98,99,100,101,102,103,104,105,106,107,108,109,110,111,112,113,114]. Eleven countries reported germline gene variant data in breast cancer (Argentina, Brazil, Chile, Colombia, Costa Rica, Cuba, Mexico, Peru, Puerto Rico, Uruguay, and Venezuela). Six hundred and ninety-two variants were found in *BRCA1/2* genes. Additionally, 126 variants were reported in 43 non-BRCA genes of patients from 7 countries. All variants were investigated in the Catalogue of Somatic Mutations in Cancer (COSMIC) (https://cancer.sanger.ac.uk/cosmic) and the ClinVar (https://www.ncbi.nlm.nih.gov/clinvar/) databases. The COSMIC database is the largest curated resource, with cancer mutation data from over 32,000 genomes including samples from The Cancer Genome Atlas (TCGA) and the International Cancer Genome Consortium (ICGC). ClinVar was used to measure the clinical significance of the reported data. 

Among variants in *BRCA1/2* genes, 202 were not found in the databases, 316 were classified as pathogenic, 59 as benign, and 115 were classified as “conflicting interpretations”, “uncertain significance”, “likely pathogenic”, and “likely benign”. Regarding the variants in non-BRCA genes, 22 variants were classified as pathogenic, 10 as benign, 57 as “conflicting interpretations”, “uncertain significance”, “likely pathogenic”, “likely benign”, “drug response”, “risk” and “risk factor”, and 37 were not found in the databases. Variants classified as “conflicting interpretations”, “uncertain significance”, “likely pathogenic”, “likely benign”, “drug response”, “risk”, and “risk factor”, were grouped as variants of unknown significance (VUS) for the purposes of this study.

## 3. Results

### 3.1. The Scope of Germline Mutations in Breast Cancer in LA Countries 

From our literature analysis, only 11 out of 21 countries documented germline data for breast cancer cases. Brazil led the list of reports (32), followed by Chile, Mexico, Colombia, and Argentina. Cuba and Venezuela only had one report each (Table 1). No germline data were found for studies performed in Ecuador, Paraguay, Panama, Dominican Republic, El Salvador, Bolivia, Guatemala, Nicaragua, Honduras, and Belize. Most of the data for germline variants in HBC accounts for the *BRCA1/2* genes and just a few studies include non-BRCA genes in a few LA countries.

### 3.2. Genes reported for HBC in LA countries

The study found 363 variants in *BRCA2* and 329 variants in *BRCA1* in 11 countries (Argentina, Brazil, Chile, Colombia, Costa Rica, Cuba, Mexico, Peru, Puerto Rico, Uruguay, and Venezuela). In addition, variants were also observed in 43 non-BRCA genes like *ATM*, *TP53*, *CHEK2*, *BARD1*, *MLH1*, *PALB2*, *BRIP1*, *MSH2*, *MSH6*, *NBN*, and *PMS2* in Chile, Brazil, Colombia, and Mexico (Table 2, Figure S1). 

### 3.3. BRCA1 and BRCA2 Genes in LA countries

Studies conducted in some LA countries have established frequent and founder *BRCA1/2* mutations in the region. The *BRCA1* 3450del4, c.5123C>A variant and *BRCA2* 3034del4 are considered founder mutations in Colombia [67,92,115]. The variants 5382insC in *BRCA1* and 6633del5 and 156_157insAlu in *BRCA2* are prevalent in Brazil [52,105,115,116]. Three variants observed in the Ashkenazi community, c.66_67delAG and c.5263insC in *BRCA1*, and c.5946delT in *BRCA2* were reported in Argentina [92,98,105]. In Mexico, the *BRCA1* ex9–12 deletion is reported as a founder mutation [88], while the variants, 2805_2808delAGAT and 3124_3133delAGCAATATTA in *BRCA1*, and 2639_2640delTG and 5114_5117delTAAA in *BRCA2* are reported as pathogenic [89,105,115]. In Puerto Rico, the variant E1308X in *BRCA2* is present in most cases of HBC [105], while in Chile, the variants c.3331_3334delCAAG and c.3759dupT in *BRCA1* and c.4740_4742dupTG, c.5146_5149delTATG in *BRCA2* are more prevalent [67]. In Peru, three recurrent mutations were described, 185delAG and 2080delA in *BRCA1*, and mutation 3034del4 in *BRCA2* have been observed [102,116]. In Costa Rica, *BRCA2* variants such as the 5531delTT are frequently reported [111,116]. No recurrent mutations in *BRCA1/2* genes were found in Venezuela [114,116]. No reports on *BRCA1/2* variants were registered for Belize, Bolivia, the Dominican Republic, Ecuador, El Salvador, Guatemala, Honduras, Nicaragua, Panama, and Paraguay. This evidence suggests that some variants are preferentially distributed in particular LA countries. 

Some other *BRCA1/2* variants are observed in more than one LA country (Table 3), like the pathogenic variant *BRCA2* c.2808_2811del variant reported in seven countries (Argentina, Brazil, Colombia, Mexico, Peru, Uruguay, and Venezuela) and the pathogenic *BRCA1* variants c.68_69delAG and c.211A>G observed in six countries. Remarkably, the *BRCA2* variant c.7469T>C classified as benign in ClinVar was observed in HBC cases in Argentina, Brazil, Colombia, Cuba, Mexico, Uruguay, and Venezuela, prompting a reconsideration of its reclassification. In summary, there are shared and specific *BRCA1/2* variants in HBC patients, reflecting the ethnic heterogeneity in Latin America [105].

There are also reports of large genomic rearrangements (LGR) in *BRCA1/2* in LA countries (Table 4). Brazil has reported 18 different LGR in *BRCA* genes, more than in other LA countries. The deletion of exons 1-2 in *BRCA1* was reported in three cases in Brazil and Puerto Rico [56,63,106]. The 6kb duplication in exon 13 in *BRCA1* was reported in seven Brazilian patients [51]. In Mexico, the exon 9–12 deletion in *BRCA1* is considered as a founder mutation that has been found in 25 cases so far [80,81,84,88]. Interestingly, the LGR g.26826_30318del in *BRCA2* found in Brazil was associated with high-risk male breast cancer [35,64]. There are also descriptions of LGRs in *BRCA1/2* in breast cancer patients from Peru, Puerto Rico, and Uruguay [104,106,109].

### 3.4. Non-BRCA Genes Reported in Breast Cancer in LA Countries

**Figure 2:**
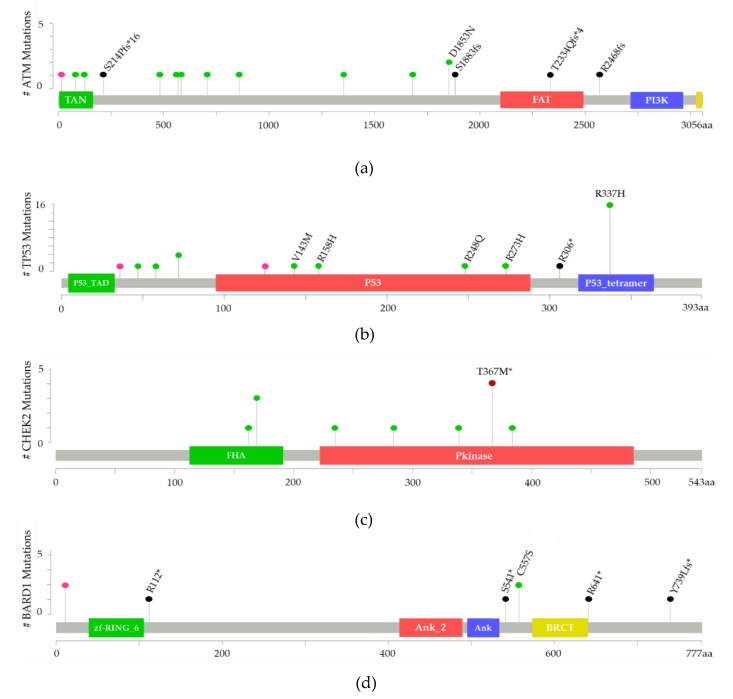
Mutations in non-BRCA genes reported in LA breast cancer patients. Frequently reported mutations are observed in *ATM*, *TP53*, *CHEK2* and *BARD1* genes. (a) *ATM* likely loss of function and therefore likely oncogenic mutations S214Pfs*16, S1883fs, T2334Qfs*4, and R2568fs; (b) *TP53* likely loss of function and therefore likely oncogenic mutations V143M, R158H, R248Q, R273H, R306*, and R337H; (c) *CHEK2* most reported mutation T367M*; (d) *BARD1* likely loss of function and therefore likely oncogenic mutations R112*, S541*, RG41* and Y739Lfs*. Mutation type: missense (green), truncating (black), in frame (red), other (pink).

Brazil, Chile, Colombia, Mexico, Peru, Puerto Rico, and Uruguay are the only LA countries that have conducted screenings for non-BRCA genes in breast cancer cases. We found 126 variants in 43 non-BRCA genes (Table 5). Variants were found in *ATM*, *TP53*, *CHEK2*, *BARD1*, *MLH1*, *PALB2,* and *BRIP1*. The most frequent variants of *ATM*, *TP53*, *CHEK2*, and *BARD1* are represented in Figure 2 as lollipop plots following published instructions [117,118]. The ataxia telangiectasia mutated (*ATM*) gene was the most reported in LA breast cancer cases. Seventeen variants were found in *ATM* in Mexico, Brazil, Chile, and Colombia, 11 variants in TP53 (Brazil, Colombia, and Uruguay) and 8 variants in CHEK2 (Brazil, Chile, Mexico, and Uruguay). Furthermore, six variants in each of the following genes were found in HBC cases: *BARD1*, *MLH1*, *PALB2* and five variants in *BRIP1* (Brazil, Chile, Colombia, Mexico, Peru, and Uruguay). The pathogenic classification of these variants is described according to ClinVar and COSMIC databases in Table S1. The *CHEK2* variant c.1100delC reported in Brazil and Chile presents conflicting interpretations of pathogenicity according to ClinVar and is not reported in the COSMIC database. The *TP53* variant c.1010G>A (p.R337H) observed in Brazil is considered to be pathogenic by ClinVar, but it is not classified as such by COSMIC [115].

**Figure 3:**
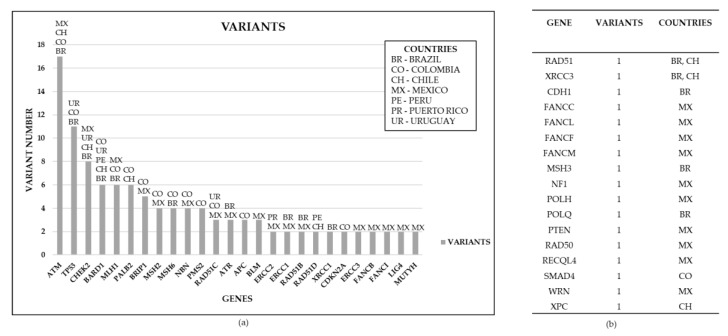
Non-BRCA gene variants reported from breast cancer patients in 7 LA countries. (a) Reported number of germline genes variants in breast cancer by country in LA; (b) Unique gene variants reported in LA countries.

Non-BRCA genes with at least two reported variants in LA countries are presented in Figure 3A. Mexico, Brazil, and Colombia are the countries with more reported variants in non-BRCA genes. Non-BRCA genes with only one variant reported in LA are described in Figure 3B. A total of 10 non-BRCA genes with one variant are reported in Mexico [81]. Additionally, only one variant was found in *RAD51*, *XRCC3*, *CDH1*, *MSH3*, *POLQ*, *SMAD4*, and *XPC* genes in Brazil, Chile, and Colombia.

Cock et al. [92] reported germline mutations in non-*BRCA* genes (*PALB2*, *ATM*, *MSH2*, *PMS2*, *MSH6,* and *TP53*) in Colombia. Among their findings, the mutation c.137G>T in *PMS2* was identified in a patient with early-onset breast cancer (31 years old), an invasive ductal carcinoma, HR-, HER2+, and positive family history. Another patient diagnosed at 36 years with invasive ductal carcinoma HR+, HER2-, had a *PALB2* mutation c.1240C>T. There was another patient diagnosed at 48 years with invasive ductal carcinoma and positive history of familial cancer carrying the *ATM* c.43del variant. VUS were also reported in patients carrying *PMS2*, *BARD1*, *RAD51C*, *BRIP1*, *MSH6*, and *MSH2* variants [92]. A previously study of Mexican patients carried out by Quezada et al. describes variants in DNA repair genes including *ATM*, *ERCC3*, *FANCI*, *ATR*, *MLH1*, *NBN*, *RAD51C*, non-BRCA genes (54%) and *BRCA1/2* (46%) [81].

## 4. Biallelic Cases, Double Heterozygosity and Young Women in LA Countries

In addition to cancer-associated variants in *BRCA1/2* and non-BRCA genes in breast cancer, there have been reports of biallelic mutations in Australia, Italy, Denmark, Spain, Korea, and other Caucasian populations [119]. Mutations in *FANCM*, *ATM*, *FANCE,* and *PALB2* contribute to locus heterogeneity, besides *BRCA1/2* [120]. Cases harboring biallelic and double heterozygosity are listed in Table 6. The study performed in Colombia by Cock et al. found cases caused by locus heterogeneity involving *BRCA1*, *MSH6*, *RAD51*, *PMS2*, *PALB2*, *BRCA2*, and *SMAD4* [92]. Interestingly, patients with double heterozygosity presented a familial cancer history and were diagnosed at early ages (before 40 years) [92]. In addition, HBC cases caused by biallelic variants in *BRCA1/2* genes affecting young patients were reported in Brazil, Mexico, and Venezuela [63,87,114]. Notoriously, a 12-year old Argentinian patient with a triple-negative breast tumor was double heterozygous for *BRCA1* and *BRCA2* variants classified as benign, although she also carried some other non-reported variants [101]. Studies in communities like those described here may help to define the clinical phenotypes of HBC cases caused by locus heterogeneity.

Nowadays, a plethora of commercial testing panels are available including “Breast Next” from Ambry Genetics, “OncoGeneDx” from GeneDx, “My Risk” from Myriad Genetics, and others. These panels along with NGS and exome analysis provide a large amount of data yet to be analyzed for breast cancer risk and its management [121]. According to the NCCN guidelines, genetic evaluation of genes like *BRCA1/2*, *ATM*, *CDH1*, *CHEK2*, *NBN*, *NF1*, and *PALB2* is recommended for HBC prevention. Other genes associated to breast cancer risk included in the NCCN guidelines are *BARD1*, *BRIP1*, *MSH2*, *MLH1*, *MSH6*, *PMS2*, *RAD51C*, and *RAD51D*, but they are not considered for breast cancer management and assessment [16]. The growing number of genes and variants associated with HBC is a challenge to the standardized systems used for clinical testing, including the re-evaluation of the variants of unknown significance (VUS). VUS are not rare, for example, there are thousands of VUS reported for *BRCA1/2* genes [122,123]. The increased number of VUS in BRCA genes correlates with the discovery of new variants in non-Caucasian ethnic groups. In individuals of European ancestry across the United States, VUS account for approximately 5–6% of the variants reported in clinical tests, and up to 21% in patients with African-American ancestry. Recent studies show a higher rate of VUS in *BRCA1/2* for non-Caucasians (36%) than in Caucasians (27%) [124]. Testing laboratories in Europe estimate that up to 15% of alterations in *BRCA1/2* are VUS [123]. Furthermore, reported variants in non-BRCA genes increase the complexity of pathogenicity classification and genetic counseling.

Information on non-BRCA gene variants in LA is scarce due to the low coverage for genetic testing in the health systems of the region, among other reasons [92,125]. This hinders the identification of new variants, their evaluation for clinical significance, and the risk management assessment.

## 5. Therapy Recommendation for DNA Repair Related Genes 

DNA double-strand breaks (DSB) are among the first procedures that take place in cancer formation and progression because of endogenous and exogenous factors [126]. With DSB, two main mechanisms may be activated during the repair process, HR and non-homologous end joining (NHEJ) [127]. Most of the non-BRCA genes frequently reported in breast cancer participate in different DNA repair pathways. Reported DSB repair variants in breast cancer comprise *PALB2*, *NBN*, *RAD51*, *ATM*, *CHEK2*, *ATR*, *RAD50*, and *WRN* [128]. Likewise, reported variants include genes participating in mismatch repair (MMR) such as *MSH2*, *MSH6*, *PMS2*, and *MLH1* [129] and genes participating in the Fanconi Anemia repair pathway like *FANCM*, *FANCI*, *FANCB*, *FANCC*, *FANCL* [130]. In addition to these pathways, variants in *XPC*, *ERCC1*, *ERCC2* and *ERCC3* genes relate to nucleotide excision repair. Variants in *XRCC1* and *MUTYH* participate in base excision repair [131]. 

DNA repair genes display high mutation incidence in cancer, when DNA repair pathways are compromised, mutation rate arises because alternative error-prone repair pathways are used by the cell [11,132]. The study of the repair pathway mechanisms has identified new targets for therapy that might be useful in some types of cancer. For instance, PARP inhibitors such as Olaparib are used for ovarian cancer treatment based on the concept of synthetic lethality and are currently being studied in breast, prostate and gastrointestinal cancers. Besides PARP, there are other key components with potential for targeted therapy; *ATR* and *ATM* are major targets for inhibition as well as *CHEK1/2* and DNA-PKs. For instance, M6620, the first inhibitor of *ATR* has been tested and *ATM* inhibitors such as M3541 are currently in clinical trials [133,134]. 

A different therapy approach that displays promising results in cancer treatment is immunotherapy with PD-1 and PD-L1 inhibitors [30,135]. High expression of PD-L1 on tumor cells or tumor-infiltrating lymphocytes (TILs) results in exhaustion of T cells and an attenuated tumor-specific immunity that promotes tumor progression [136]. Diverse studies show a possible relationship between altered DNA repair pathways that increase the mutational burden and immunotherapy response. For example, colorectal cancer patients with altered MMR pathways display high microsatellite instability, expression of PD-L1, as CD3+, CD8+ TILs and tumor-associated macrophages located at invasive fronts of the tumor. The mutational burden was associated with mutations in *ATM*, MMR deficiency, and therefore loss of expression in *MLH1*, *MSH2*, *MSH6,* and *PMS2* [33]. In addition, patients with tumor DNA repair deficiencies such as *POLE* and *POLD* mutations, are considered good candidates for checkpoint immunotherapy [137,138]. In non-small cell lung cancer (NSCLC), a study by Chae et al. [28] showed that tumors with altered HR genes, MMR genes or *POLE* contained higher mutational load than tumors with wildtype DNA repair genes. These tumors also contained higher infiltration of T cells and other cells that perform anti-tumor activity. The best treatment responses were observed in patients with high mutational burden with PD-1 inhibitors. The group of better responder patients displayed a neoantigen load and mutations in *POLE*, *POLD1,* and *MSH2*. Based on the premise that DNA repair loss results in elevated anti-tumor immune response, improved clinical outcomes were observed in patients with DNA repair gene mutations. Therefore, mutations in DNA repair genes in lung cancer were linked to increased TILs as CD4+ and CD8+ in the tumor. For patients with higher mutational burden, there is a greater likelihood of the formation of immunogenic epitopes expressed only in cancerous cells [28]. Similarly, in high-grade ovarian cancer altered *BRCA1/2* results in a higher mutational load, therefore, tumors harbor more tumor-specific neoantigens, increased TILs including CD3+ and CD8+ and PD-1 and PD-L1 expression [29,136]. Strickland et al. found that tumors with altered HR repair show higher neoantigens along with improved overall survival. They inferred that high grade serous ovarian cancer with mutations in *BRCA1/2* may be more sensitive to immune checkpoint inhibitors PD-1 and PD-L1 in comparison with tumors proficient in HR repair [29].

Breast cancer patients with compromised DNA repair mechanisms display high-risk tumor characteristics such as changes in cell morphology that promote invasion [139]. When HR is compromised, alternative error-prone DNA repair pathways are used and thus there is a chance that errors will occur, such as indels in the cell [140,141]. There are frequently mutated DNA repair genes in breast cancer and their analysis is fundamental to determine the tumor phenotype and clarify if a high mutational load is due to deficient DNA repair performance. PARP inhibitors are approved for some breast cancer cases, Olaparib was approved by the FDA for germline BRCA-mutated metastatic breast cancer on January 12, 2018 [142]. Similarly, Talazoparib was approved for germline BRCA-mutated HER2-negative locally advanced or metastatic breast cancer by the FDA in October, 2018 [143]. Moreover, several studies in Clinical Trials (https://clinicaltrials.gov/ct2/home) are focused on DNA repair genes. For instance, the study NCT03495544 focuses on the association between germline DNA repair genes mutations and PD-L1 expression level in breast cancer. Studies for PARP inhibitors such as Rucaparib have been completed, studies for Niraparib are still recruiting for Phase I and Phase III trials, and BMN673 is being tested in advanced breast cancer. On the other hand, immunotherapy studies in breast cancer like the one conducted by Schmid et al. [144] for triple-negative breast cancer (TNBC) showed that the combination of PD-L1 inhibitor Atezolizumab with nab-paclitaxel resulted in improvement in overall survival with a median of 9.5-month (HR 0.62, 95% CI 0.45–0.86) in patients with PD-L1 positive immune infiltration [144]. Another combined therapy for TNBC of Pembrolizumab an anti-PD-1 with chemotherapy increased the pathological complete response rates [145]. The potential for immunotherapy in breast cancer is promising, suggesting combination therapies for future clinical trials [146].

Even though novel cancer treatment strategies are appearing, in Latin America the availability of these therapies is challenging [147]. Over the last five years, about 64% of medicine newly released to the market was sold exclusively in the US, 24% of these new drugs were sold in Western Europe and 7% in Japan. Therefore, the remaining 5% is distributed in the rest of the world [147]. Between 2009 and 2013, 37 new cancer drugs were launched worldwide and only 17 of them are available in Mexico and 10 in Brazil [148], and access to high-cost cancer drugs is a barrier in the LA region [149]. In LA countries, use of novel drugs differs widely by insurance type, therefore, one promising solution to improve access to these therapies is participation in clinical research [148,149]. For breast cancer, chemotherapy with anthracyclines is accepted in the region, however, targeted therapy drugs such as Trastuzumab is not accessible to all patients. Restrictions to access new drugs leaves patients with few therapeutic alternatives, disease progression, and consequently, poor outcomes [150]. Therefore, one option for new drugs to be available in LA countries is through participation in clinical trials. Some of these countries are currently participating in clinical trials that are focused on PARP inhibitors and immune checkpoint inhibitors. Velaparib, Talazoparib, and Olaparib are PARP inhibitors and currently, active clinical trials NCT01506609, NCT02595905, and NCT02163694 are testing Velaparib in combination with Carboplatin, Paclitaxel, and Cisplatin in breast cancer patients harboring BRCA mutations in Argentina, Brazil, Chile, Colombia, Puerto Rico, Mexico. Similarly, a phase 3 study NCT01945775 in Brazil is currently testing Talazoparib in patients with metastatic breast cancer and BRCA mutations. In addition, Olaparib is currently being tested in metastatic breast cancer patients with germline *BRCA1/2* mutation in Peru and Mexico in a phase 3 study (NCT02000622). Also, some immunotherapy agents are currently in clinical trials in Latin America countries. Pembrolizumab, a PD-1 inhibitor, is being tested (NCT02447003) in metastatic TNBC in Puerto Rico. Pembrolizumab in combination with chemotherapy (NCT03036488) is being tested in Colombia and Brazil. Clinical trial NCT03797326 is studying Pembrolizumab in combination with Lenvatinib in Chilean TNBC patients. Clinical trial NCT03725059 is currently recruiting patients in Brazil and Colombia to test neoadjuvant chemotherapy in combination with Pembrolizumab in early stage ER positive, HER2 negative breast cancer. The PD-1 inhibitor Nivolumab is being tested in combination with Daratumab in TNBC patients in Puerto Rico. Atezolizumab a PD-L1 inhibitor, is being tested as an immunotherapy strategy in phase 3 clinical trial NCT02425891 in combination with Nab-Paclitaxel in metastatic TNBC in Mexico, Colombia, Brazil, Argentina, and Chile. Similarly, clinical trials NCT03498716, NCT03197935, and NCT03125902 are recruiting TNBC patients in Mexico, Peru, Brazil, and Argentina to test Atezolizumab in combination with chemotherapy, Anthracyclines, Taxanes, Paclitaxel.

Based on the reported mutation data for LA countries, we consider that breast cancer patients might benefit from novel targeted therapies like PARP-inhibitors and immunotherapy. In the same way, patients with germline mutations in DNA repair genes like *PALB2*, *NBN*, *RAD51*, *ATM*, *CHEK2*, *ATR,* and *RAD50*, might benefit from PD-L1 inhibitors and PARP inhibitors. In Latin America countries, germline mutations in BRCA and non-BRCA genes have been reported mostly in patients with early onset, advanced disease or TNBC. With this in mind, PARP inhibitors and immunotherapy might be a good strategy for breast cancer patients harboring these previously mentioned mutations. Consequently, further studies focusing on DNA repair gene mutations and their role as novel predictive markers are needed for immunotherapy response and targeted therapy in the DNA damage response (DDR) pathway [28]. We hypothesize that breast cancer patients in Latin America that have mutations in the DNA repair pathway could benefit from these kinds of therapies.

In this study, several *BRCA1/2* variants that are considered susceptible to PARP inhibitors were found according to the JAX Clinical Knowledgebase (JAX-CKB) (https://ckb.jax.org) and the Clinical Interpretations of Variants in Cancer database (https://civicdb.org/home). In these databases, seventeen variants in each *BRCA1* and *BCR2* genes are considered to be sensitive to PARP inhibitors due to loss of function or predicted loss of function. These variants were reported in some LA countries (Argentina, Brazil, Chile, Colombia, Costa Rica, Mexico, Peru, Uruguay, and Venezuela). Interestingly, some variants such as c.211A>G and c.68_69delAG in *BRCA1* were each reported in six countries (Argentina, Brazil, Chile, Colombia, Mexico, Peru, and Uruguay); and for *BRCA2*, variant c.5946delT was observed in five countries (Argentina, Brazil, Chile, Costa Rica, and Peru).

For non-BRCA genes like *ATM*, PARP inhibitors are suggested for inactivating mutations or loss of function variants; in the same way as for *BARD1*, *CHEK2*, *MSH2*, *NBN*, and *PALB2* genes. Consequently, we consider that patients in different LA countries harboring these variants could benefit from PARP inhibitors therapies.

In this work, even though rare variants are often reported and counted as mutations, most of them have not been tested in functional analysis. Herein, variants that have been reported in at least two countries for breast cancer are included, suggesting that they may have biological significance.

## 6. Conclusions

Understanding germline mutations in BRCA and non-BRCA genes in Latin American communities is necessary to improve screening strategies and to implement and develop viable precision medicine practices. This study shows that more information and analyses are required to define the prevalence of gene variants involved in HBC in LA, to define their pathogenicity and for reclassifying VUS and variants of conflicting interpretation in this population. This information will facilitate the implementation of HBC screening programs and targeted therapies in Latin America, particularly of those treatments that address DNA repair mechanisms. 

The importance of more breast cancer studies, including non-BRCA genes, is to analyze the DNA repair capacity status for a better understanding of the relationship between DNA repair and breast cancer tumor aggressivity, potential biomarkers for prognosis along with immunotherapy recommendations and possible novel targets in DDR. Therefore, patients with mutations in DNA repair genes might also be candidates for targeted therapy in order to improve their outcome. Future studies focusing on non-BRCA genes, mainly DNA repair genes, will be of great benefit for LA breast cancer patients. 

## 7. Take Home Messages

- *BRCA1/2* are the most analyzed and studied genes in LA countries, few studies report non-BRCA gene status in breast cancer.
- In addition to *BRCA1/2*, non-BRCA genes provide information about the DNA repair capacity status.
- Targeted therapy such as immunotherapy and mainly PARP inhibitors are focused on DNA repair gene status for better response.
- Studies focusing on non-BRCA genes are needed in LA countries.

## Figures and Tables

**Table 1:** BRCA and Non-BRCA genes papers in Latin America (LA).

	Country	Total Retrieved Papers ^1^	Germline Data ^2^	BRCA1/2 Papers	Non-BRCA Papers	Total Papers	References
1	Brazil	3290	✔	13	19	32	[34,35,36,37,38,39,40,41,42,43,44,45,46,47,48,49,50,51,52,53,54,55,56,57,58,59,60,61,62,63,64,65]
2	Chile	455	✔	7	7	14	[66,67,68,69,70,71,72,73,74,75,76,77,78,79]
3	Mexico	2014	✔	8	4	12	[80,81,82,83,84,85,86,87,88,89,90,91]
4	Colombia	274	✔	5	1	6	[92,93,94,95,96,97]
5	Argentina	893	✔	4		4	[98,99,100,101]
6	Peru	161	✔	2	1	3	[102,103,104]
7	Puerto Rico	253	✔	2	1	3	[105,106,107]
8	Uruguay	126	✔	3		3	[108,109,110]
9	Costa Rica	56	✔	2		2	[111,112]
10	Cuba	142	✔	1		1	[113]
11	Venezuela	76	✔	1		1	[114]
12	Ecuador	42					
13	Paraguay	31					
14	Panama	23					
15	Dominican Republic	18					
16	El Salvador	16					
17	Bolivia	13					
18	Guatemala	8					
19	Nicaragua	7					
20	Honduras	2					
21	Belize	1					
	Total	7901	11	48	33	81	

^1^ First search combining terms “Breast cancer” versus 21 LA countries (“Argentina”, “Belize”, “Bolivia”, “Brazil”, “Chile”, “Colombia”, “Costa Rica”, “Cuba”, “Dominican Republic”, “Ecuador”, “El Salvador”, “Guatemala”, “Honduras”, “Mexico”, “Nicaragua”, “Panama”, “Paraguay”, “Peru”, “Puerto Rico”, “Uruguay”, “Venezuela”); as well as “BRCA1 and BRCA2”. ^2^ Second search including papers with germline mutation data and breast cancer.

**Table 2:** Breast Cancer gene variants reported by LA countries in breast cancer.

Gene	Number of Variants	Argentina	Brazil	Chile	Colombia	Costa Rica	Cuba	Mexico	Peru	Puerto Rico	Uruguay	Venezuela
BRCA2	363	✔	✔	✔	✔	✔	✔	✔	✔	✔	✔	✔
BRCA1	329	✔	✔	✔	✔	✔	✔	✔	✔	✔	✔	✔
ATM	17		✔	✔	✔			✔				
TP53	11		✔		✔						✔	
CHEK2	8		✔	✔				✔			✔	
BARD1	6		✔	✔	✔				✔		✔	
MLH1	6		✔		✔			✔				
PALB2	6			✔	✔							
BRIP1	5				✔			✔				
MSH2	4				✔			✔				
MSH6	4		✔		✔							
NBN	4				✔			✔				
PMS2	4				✔							
APC	3				✔							
ATR	3		✔					✔				
BLM	3							✔				
RAD51C	3				✔			✔			✔	
CDKN2A	2				✔							
ERCC1	2		✔					✔				
ERCC2	2		✔					✔		✔		
ERCC3	2							✔				
FANCB	2							✔				
FANCI	2							✔				
LIG4	2							✔				
MUTYH	2							✔				
RAD51B	2		✔					✔				
RAD51D	2			✔					✔			
XRCC1	2		✔					✔				
CDH1	1		✔									
FANCC	1							✔				
FANCF	1							✔				
FANCL	1							✔				
FANCM	1							✔				
MSH3	1		✔									
NF1	1							✔				
POLH	1							✔				
POLQ	1		✔									
PTEN	1							✔				
RAD50	1							✔				
RAD51	1		✔	✔								
RECQL4	1							✔				
SMAD4	1				✔							
WRN	1							✔				
XPC	1			✔								
XRCC3	1		✔	✔								

**Table 3:** Frequent BRCA1/2 gene variants in LA.

Gene	rs	Exon	Argentina	Brazil	Chile	Colombia	Costa Rica	Cuba	Mexico	Peru	Puerto Rico	Uruguay	Venezuela
**BRCA1 VARIANT**													
c.68_69delAG	rs386833395	2	(4)	(4)	(2)	(1)			(1)	(1)			
c.181T>G	rs28897672	5	(1)	(4)	(1)								
c.211A>G	rs80357382	5	(2)	(3)	(1)				(3)	(1)		(1)	
c.3113A>G	rs16941	11	(1)	(3)	(2)					(1)			
c.3548A>G	rs16942	11	(1)	(3)					(1)	(1)			
c.1067A>G	rs1799950	11	2	(3)						(1)			
c.3119G>A	rs4986852	11	(1)	(3)	(1)				(1)				(1)
c.2612C>T	rs799917	11	(1)	(3)						(1)			
c.3331_3334delCAAG	rs80357701	11		(9)	(3)	(6)							
c.4308C>T	rs1060915	13	(1)	(2)								(1)	
c.4837A>G	rs1799966	16	(1)	(3)	(1)					(1)			
c.5123C>A	rs28897696	18	(1)	(2)		(8)			(3)				
c.5266dupC	rs397507247	20	(4)	(16)	(1)						(1)		
**BRCA2 VARIANT**													
c.865A>C	rs766173	10	(1)	(2)	(1)					(1)			
c.2971A>G	rs1799944	11	(1)	(2)	(2)				(1)	(1)			
c.5744C>T	rs4987117	11	(1)	(2)	(2)				(1)	(1)			
c.2808_2811del	rs80359351	11	(3)	(5)		(2)			(1)	(2)		(1)	(1)
c.5351dupA	rs80359507	11	(1)	(2)								(2)	
c.5946delT	rs80359550	11	(5)	(4)	(1)		(4)			(1)			
c.7469T>C	rs11571707	15	(2)	(4)		(1)		(1)	(1)			(1)	(1)
c.10234A>G	rs1801426	27	(1)	(3)					(2)				(1)

() Number of reporting papers.

**Table 4:** Large genomic rearrangements in BRCA1/2.

Gene	Mutation	Cases	Brazil	Colombia	Mexico	Peru	Puerto Rico	Uruguay	References
BRCA1	exon 1-2 deletion	3	✔				✔		[60,63,106]
BRCA1	exon 3 deletion	2	✔						[60,63]
BRCA1	exon 4-6 deletion	2	✔						[60,63]
BRCA1	exon 5-7 deletion	2	✔						[53,60]
BRCA1	exon 8 deletion	2	✔						[60,63]
BRCA1	exon 8-13 deletion	1				✔			[104]
BRCA1	exon 9-11 deletion	1			✔				[80]
BRCA1	exon 9-12 deletion	25			✔				[80,81,84,88]
BRCA1	exon 9-19 deletion	2	✔						[41,60]
BRCA1	exon 12 deletion	1			✔				[80]
BRCA1	exon 14 deletion	1						✔	[109]
BRCA1	exon 14-16 deletion	1	✔						[60]
BRCA1	exon 16-17 deletion	2	✔						[60,65]
BRCA1	exon 18-19 deletion	2	✔			✔			[60,104]
BRCA1	exon 19 deletion	1	✔						[60]
BRCA1	exon 21-23 deletion	1	✔						[60]
BRCA1	exon 24 duplication	1	✔						[65]
BRCA1	6-KB DUP EX13	7	✔						[51]
BRCA2	exon 1 deletion	3			✔				[80]
BRCA2	exon 1-14 deletion	2		✔					[94]
BRCA2	exon 2 deletion	1	✔						[60]
BRCA2	exon 11 deletion	1			✔				[80]
BRCA2	exon 13 deletion	1	✔						[60]
BRCA2	exon 14 deletion	1	✔						[60]
BRCA2	exon 15-16 deletion	1		✔					[92]
BRCA2	exon 17 deletion	1			✔				[80]
BRCA2	exon 22-24 deletion	2			✔				[80]
BRCA2	exon 23 deletion	2			✔				[80]
BRCA2	exon 25 deletion	1	✔						[60]
BRCA2	exon 26 deletion	1			✔				[80]
BRCA2	g.26826_30318del	2	✔						[35,64]

**Table 5:** Frequent variants in non-BRCA genes in LA.

Gene	rs	Variant	COSMIC	CLINVAR	Brazil	Chile	Colombia	Mexico	Peru	Uruguay
ATM	NA	c.634delT	✔		x					
ATM	NA	c.5648_5655del						x		
ATM	rs145119475	c.4060C>A		✔				x		
ATM	rs1800056	c.2572T>C	✔	✔		x				
ATM	rs1801516	c.5557G>A	✔	✔		x		x		
ATM	rs1801673	c.5558A>T	✔	✔		x				
ATM	rs200381392	c.1703G>T		✔			x			
ATM	rs202173660	c.1444A>C	✔	✔			x			
ATM	rs2234997	c.378T>A	✔	✔		x				
ATM	rs2235006	c.1744T>C		✔		x				
ATM	rs4986761	c.2119T>C	✔	✔		x				
ATM	rs587782153	c.5039C>T	✔	✔			x			
ATM	rs758962678	c.241A>G		✔				x		
ATM	rs759965045	c.7702_7703del		✔				x		
ATM	rs771887195	c.43del		✔			x			
ATM	rs786203421	c.7000_7003delTACA		✔	x					
ATM	rs786204433	c.5644C>T	✔	✔	x					
TP53	rs1042522	c.215C>G	✔	✔	x					
TP53	rs11540652	c.743G>A	✔	✔						x
TP53	rs121912664	c.1010G>A	✔	✔	x					
TP53	rs121913344	c.916C>T	✔	✔						x
TP53	rs144386518	c.173C>G	✔	✔			x			
TP53	rs1800370	c.108G>A	✔	✔	x					
TP53	rs1800371	c.139C>T	✔	✔			x			
TP53	rs28934576	c.818G>A	✔	✔						x
TP53	rs55863639	c.375G>A	✔	✔						x
TP53	rs587782144	c.473G>A	✔	✔						x
TP53	rs587782620	c.427G>A	✔	✔	x					
CHEK2	NA	c.1015C>T						x		
CHEK2	NA	c.1151delT						x		
CHEK2	NA	c.705A>C						x		
CHEK2	NA	c.852G>T						x		
CHEK2	rs1555926890?	c.506T>C						x		
CHEK2	rs555607708	c.1100delC		✔	x	x				
CHEK2	rs587781652	c.485A>G		✔	x					
CHEK2	rs864622149	c.846+1G>C		✔						x
BARD1	NA	c.2215dupT			x					
BARD1	rs143914387	c.33G>T					x			
BARD1	rs28997576	c.1670G>C				x				
BARD1	rs587781948	c.1921C>T								x
BARD1	rs758972589	c.334C>T	✔	✔					x	
BARD1	rs777937955	c.1622C>A							x	
MLH1	NA	c.1966C>T						x		
MLH1	NA	c.413A>G						x		
MLH1	NA	c.791G>A						x		
MLH1	rs148317871	c.2213G>A		✔			x			
MLH1	rs63751615	c.676C>T		✔				x		
MLH1		del_exon8	NA	NA	x					
PALB2	NA	c.1861C>A				x				
PALB2	NA	c.483C>G					x			
PALB2	rs150390726	c.23C>T		✔			x			
PALB2	rs152451	c.1676A>G		✔		x				
PALB2	rs180177100	c.1240C>T	✔	✔			x			
PALB2	rs45551636	c.2993C>T				x				
BRIP1	rs202072866	c.415T>G	✔	✔				x		
BRIP1	rs28997569	c.790C>T		✔			x			
BRIP1	rs371185409	c.3079G>A		✔			x			
BRIP1	rs45589637	c.2220G>T		✔			x			
BRIP1	rs759031349	c.689C>T		✔				x		

✔Databases; **x** Country; **NA:** Not available.

**Table 6:** Biallelic and locus heterogeneity mutations reported in LA breast cancer cases.

Patient ID	Gene 1	Gene 2	Age Onset	Country	Family History	Subtype	Reference
NA	BRCA1: c.1674del (pathogenic)	MSH6: c.2419G>A (uncertain significance)	NA	Colombia	YES	NA	[92]
NA	BRCA1: c.1674del (pathogenic)	PMS2: c.2395C>T (uncertain significance); RAD51C: c.492T>G (uncertain significance)	NA	Colombia	YES	NA	[92]
15	PALB2: c.1240C>T (pathogenic)	PMS2: c.241G>A (uncertain significance)	36	Colombia	YES	ER+, HER2-, invasive ductal carcinoma	[92]
8	BRCA2: c.5616-5620del (not reported)	SMAD4: c.677C>T (conflicting interpretations)	35	Colombia	YES	HR+, HER2- invasive ductal carcinoma	[92]
NA	BRCA1: c.4357+1G>T (pathogenic)	BRCA2: c.6405_6409delCTTAA (pathogenic)	38	Brazil	NA	ipsilateral BC	[63]
NA	BRCA1: LGR (deletion of exons 4–6)	BRCA2: c.9004G>A (conflicting interpretation)	43	Brazil	NA	NA	[63]
NA		BRCA2: c.8878C>T (pathogenic), c.9699_9702delTATG (pathogenic)	52	Brazil	NA	NA	[63]
CM001	BRCA1: c.1129_1135insA (not reported), c.4063_4065delAAT (conflicting interpretations)		37	Venezuela	YES	ER+, PR+	[114]
CM055	BRCA1: c.1129_1135insA (not reported), c.4063_4065delAAT (conflicting interpretations)		48	Venezuela	YES	ER-, PR-	[114]
CM031		BRCA2: c.1282T>C (not reported), c.3479G>A (conflicting interpretations)	49	Venezuela	YES	NA	[114]
5	BRCA2: c.865A>C (benign), c.2971A>G(benign), c.8851G>A (benign)		33	Mexico	NA	ductal	[87]
12	BRCA1: c.2245G>T (uncertain significance)	BRCA2: p.Ile3412Val (benign)	34	Mexico	NA	ductal	[87]
7	BRCA1: c.442-34C>T (benign)	BRCA2: c.865A>C (benign), c.2971A>G (benign)	34	Mexico	NA	ductal	[87]
17	BRCA1: c.3548A>G (benign), c.442-34C>T (benign)		30	Mexico	YES	ER+, PR+, HER2-	[89]
A11	BRCA1: c.4308T>C (benign), c.442-34C (not reported), c.5152+66G>A (benign), c.548-58delT (benign)	BRCA2: c.426+67A>C (not reported), c.426-89T>C (benign), c.7435+53C>T (benign)	12	Argentina	YES	TNBC, Secretory carcinoma	[101]
A17	BRCA1: c.4308T>C (benign), c.5152+66G>A (benign), c.548-58delT (benign)		25	Argentina	NO	PR+, ER+, HER2+, infiltrating ductal carcinoma	[101]
A18	BRCA1: c.442-34T>C (not reported)	BRCA2: c.7469T>C (benign), c.681+56C>T (benign), c.7242A>G (benign)	21	Argentina	NO	ER+, PR+ HER2-, infiltrating lobular carcinoma	[101]

() Pathogenicity: ClinVar; NA: Not Available; LGR: Large Genomic Rearrangements.

## Supplementary Materials

The following are available online at https://www.mdpi.com/2073-4425/10/10/786/s1, Figure S1: *BRCA1/2* and non-BRCA gene variants reported from breast cancer cases in LA countries, Table S1: Frequent variants in non-BRCA genes in LA and their pathogenic classification.
