# Supplementary material for: Landscape of Germline Mutations in DNA Repair Genes for Breast Cancer in Latin America: Opportunities for PARP-Like Inhibitors and Immunotherapy
Source: Genes (Basel). 2019 Oct 10;10(10):786. doi: 10.3390/genes10100786 (PMC6827033; doi:10.3390/genes10100786)
Supplement: Supplementary file 1 [file genes-10-00786-s001.zip › Urbina-Jara Laura Keren et al-Supplementary figure S1-Genes Journal-10-09-19.docx]

BRCA1/2 GENE VARIANTS NON-BRCA GENE VARIANTS


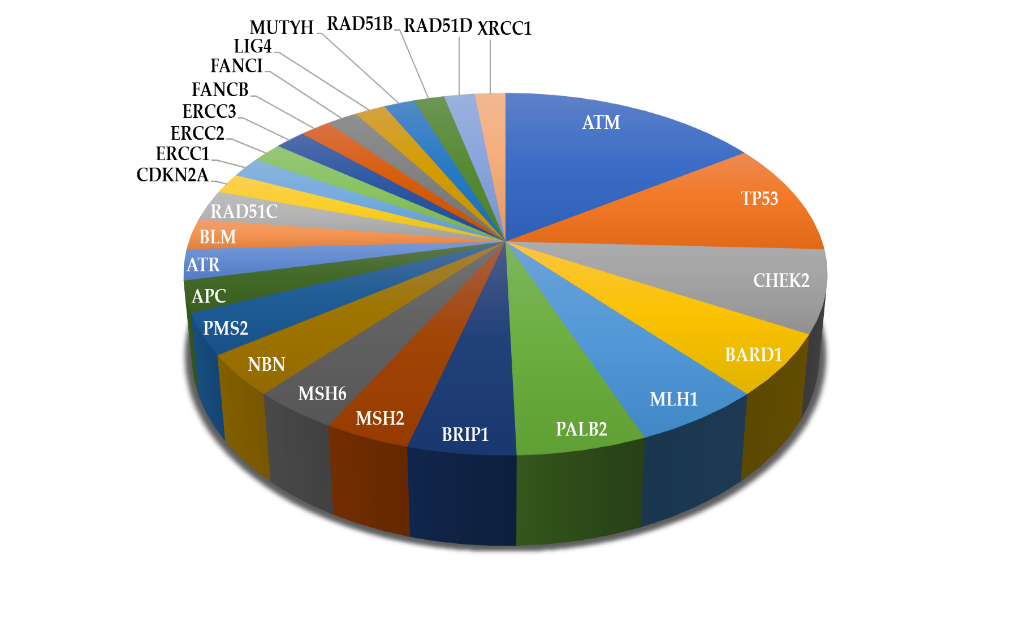

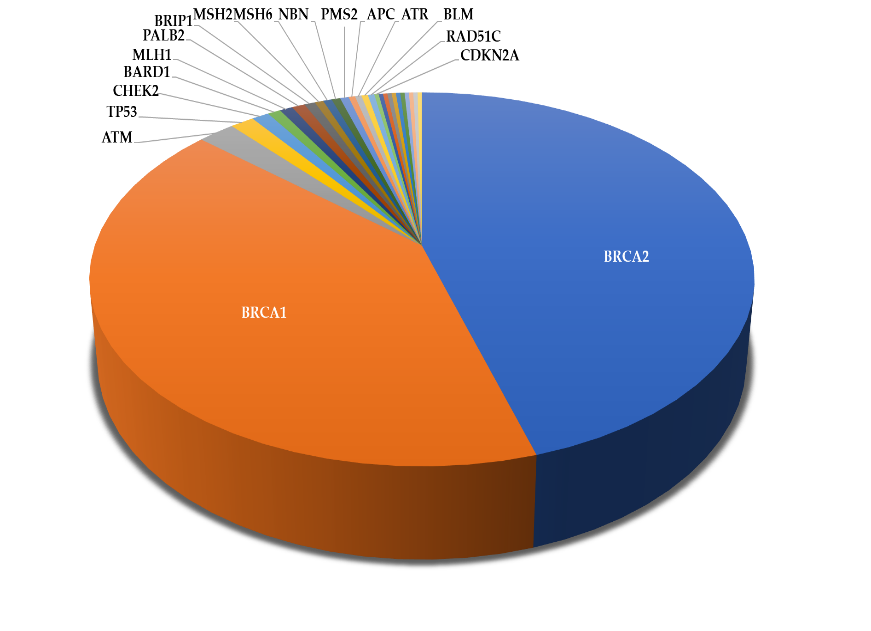


Figure S1. *BRCA1/2* and non-BRCA gene variants reported from breast cancer cases in LA countries. Based in 81 studies published between 2000 and April 5, 2019. (a) BRCA1/2 and non-BRCA gene variants; (b)Non-BRCA gene variants.
