# Supplementary material for: Landscape of Germline Mutations in DNA Repair Genes for Breast Cancer in Latin America: Opportunities for PARP-Like Inhibitors and Immunotherapy
Source: Genes (Basel). 2019 Oct 10;10(10):786. doi: 10.3390/genes10100786 (PMC6827033; doi:10.3390/genes10100786)
Supplement: Supplementary file 1 [file genes-10-00786-s001.zip › Urbina-Jara Laura Keren et al-Supplementary table S1-Genes Journal-10-09-19.docx]

**Table S1.** Frequent variants in non-BRCA genes in LA and their pathogenic classification

| **Gene** | **rs** | **Variant** | **COSMIC** | **CLINVAR** |
| --- | --- | --- | --- | --- |
| ATM | NA | c.634delT | NA | Not reported |
| ATM | NA | c.5648_5655del | Not reported | Not reported |
| ATM | rs145119475 | c.4060C>A | Not reported | Conflicting interpretations |
| ATM | rs1800056 | c.2572T>C | Pathogenic (score 0.73) | Conflicting interpretations |
| ATM | rs1801516 | c.5557G>A | Pathogenic (score 0.98) | Benign |
| ATM | rs1801673 | c.5558A>T | Pathogenic (score 0.97) | Conflicting interpretations |
| ATM | rs200381392 | c.1703G>T | Not reported | Conflicting interpretations |
| ATM | rs202173660 | c.1444A>C | Pathogenic (score 0.94) | Uncertain significance |
| ATM | rs2234997 | c.378T>A | Neutral (score 0.18) | Benign |
| ATM | rs2235006 | c.1744T>C | Not reported | Conflicting interpretations |
| ATM | rs4986761 | c.2119T>C | Neutral (score 0.15) | Benign |
| ATM | rs587782153 | c.5039C>T | Pathogenic (score 0.99) | Benign |
| ATM | rs758962678 | c.241A>G | Not reported | Uncertain significance |
| ATM | rs759965045 | c.7702_7703del | Not reported | Pathogenic |
| ATM | rs771887195 | c.43del | Not reported | Pathogenic |
| ATM | rs786203421 | c.7000_7003delTACA | Not reported | Pathogenic |
| ATM | rs786204433 | c.5644C>T | Pathogenic (score 0.74) | Pathogenic |
| TP53 | rs1042522 | c.215C>G | Neutral (score 0.36) | Drug response |
| TP53 | rs11540652 | c.743G>A | Pathogenic (score 0.98) | Pathogenic |
| TP53 | rs121912664 | c.1010G>A | NA | Pathogenic |
| TP53 | rs121913344 | c.916C>T | Pathogenic (score 0.70) | NA |
| TP53 | rs144386518 | c.173C>G | Pathogenic (score 0.99) /Neutral (score 0.28) | Conflicting interpretations |
| TP53 | rs1800370 | c.108G>A | Neutral (score 0.02) | Benign |
| TP53 | rs1800371 | c.139C>T | Neutral (score 0.02) | Conflicting interpretations |
| TP53 | rs28934576 | c.818G>A | Pathogenic (score 1.00) | Pathogenic |
| TP53 | rs55863639 | c.375G>A | NA | Pathogenic |
| TP53 | rs587782144 | c.473G>A | Pathogenic (score 0.99) | Pathogenic |
| TP53 | rs587782620 | c.427G>A | Pathogenic (score 0.83) | Conflicting interpretations |
| CHEK2 | NA | c.1015C>T | Not reported | Not reported |
| CHEK2 | NA | c.1151delT | Not reported | Not reported |
| CHEK2 | NA | c.705A>C | Not reported | Not reported |
| CHEK2 | NA | c.852G>T | Not reported | Not reported |
| CHEK2 | rs1555926890? | c.506T>C | Not reported | Not reported |
| CHEK2 | rs555607708 | c.1100delC | Not reported | Conflicting interpretations |
| CHEK2 | rs587781652 | c.485A>G | Not reported | Uncertain significance |
| CHEK2 | rs864622149 | c.846+1G>C | Not reported | Pathogenic |
| BARD1 | NA | c.2215dupT | Not reported | Not reported |
| BARD1 | rs143914387 | c.33G>T | Not reported | Not reported |
| BARD1 | rs28997576 | c.1670G>C | Not reported | Not reported |
| BARD1 | rs587781948 | c.1921C>T | Not reported | Not reported |
| BARD1 | rs758972589 | c.334C>T | Pathogenic (score 0.86) | Pathogenic |
| BARD1 | rs777937955 | c.1622C>A | Not reported | Not reported |
| MLH1 | NA | c.1966C>T | Not reported | Not reported |
| MLH1 | NA | c.413A>G | Not reported | Not reported |
| MLH1 | NA | c.791G>A | Not reported | Not reported |
| MLH1 | rs148317871 | c.2213G>A | Not reported | Uncertain significance |
| MLH1 | rs63751615 | c.676C>T | Not reported | Pathogenic |
| MLH1 |  | del_exon8 | NA | NA |
| PALB2 | NA | c.1861C>A | Not reported | Not reported |
| PALB2 | NA | c.483C>G | Not reported | Not reported |
| PALB2 | rs150390726 | c.23C>T | Not reported | Conflicting interpretations |
| PALB2 | rs152451 | c.1676A>G | Not reported | Likely benign |
| PALB2 | rs180177100 | c.1240C>T | Pathogenic (score 0.97) | Pathogenic |
| PALB2 | rs45551636 | c.2993C>T | Not reported | Not reported |
| BRIP1 | rs202072866 | c.415T>G | Pathogenic (0.90) | Uncertain significance |
| BRIP1 | rs28997569 | c.790C>T | Not reported | Likely benign |
| BRIP1 | rs371185409 | c.3079G>A | Not reported | Conflicting interpretations |
| BRIP1 | rs45589637 | c.2220G>T | Not reported | Conflicting interpretations |
| BRIP1 | rs759031349 | c.689C>T | Not reported | Conflicting interpretations |

NA: Not Available

Functional Analysis through Hidden Markov Models (FATHMM)

() FATHMM prediction of the functional consequences of coding and non-coding variants
